# Prevalence of Dental Caries, Oral Hygiene Knowledge, Status, and Practices among Visually Impaired Individuals in Chennai, Tamil Nadu

**DOI:** 10.1155/2017/9419648

**Published:** 2017-03-28

**Authors:** James Rufus John, Breena Daniel, Dakshaini Paneerselvam, Ganesh Rajendran

**Affiliations:** ^1^School of Science and Health, Western Sydney University, Campbelltown, NSW, Australia; ^2^Department of Public Health Dentistry, Priyadarshini Dental College and Hospital, Tamil Nadu, India

## Abstract

*Aim*. To assess the prevalence of dental caries, oral hygiene knowledge, status, and practices among visually impaired individuals in Chennai, Tamil Nadu.* Materials and Methods*. A cross-sectional study was conducted among 404 visually impaired individuals in Chennai city, Tamil Nadu. Four schools were randomly selected for conducting the study. The oral hygiene status, prevalence of caries, and knowledge and attitude towards oral care among visually impaired individuals were collected and analysed.* Results*. In the present study, whilst 42% of individuals had fair oral hygiene status, 33% had good hygiene followed by 25% having poor oral hygiene. The overall mean number of DMFT was estimated to be 4.5 ± 2.7. The mean number of decayed teeth was 3.1 ± 2.2, mean number of missing teeth was 0.8 ± 1.4, and mean number of filled teeth was 0.5 ± 1.3.* Conclusion*. Whilst oral hygiene status was found to be relatively fair, there was a high rate of dental caries among the sample population. This shows that there is lack of knowledge regarding oral health maintenance. Therefore, early identification of caries coupled with effective oral health promotion programs providing practical knowledge to visually impaired students would prove beneficial.

## 1. Introduction

Oral health has a great impact on the overall health and well-being of an individual. Maintenance of oral health is particularly challenging in people with special needs. “Special needs” is a term used in clinical diagnostic and functional development to describe individuals who require assistance for disabilities that may be medical, mental, or psychological [1]. Among various categories of individuals with special needs, visually impaired people face greater challenge surviving day to day. The World Health Organization (WHO) defines blindness as “visual acuity of less than 3/60 m or corresponding visual field loss in the better eye with the best possible correction” meaning that whilst a visually impaired person could see three meters, a nonvisually impaired person could see 60 meters [2]. Blindness may be either complete or partial, which is congenital or acquired. According to the Indian sample survey of 2011, about 18.8% are visually impaired followed by disability in speech, hearing, and movement and being mentally handicapped. The 2011 census in India identifies 26,810,557 disabled citizen representing 2.21% of the population [3].

Most of the visually impaired individuals require the help of others to accomplish their task round the clock. The amplitude of oral health problem in disabled people is worse when compared to general population and disabled people have more untreated dental diseases and problems in accessing dental care [4]. Dental caries is a common unmet problem among disabled population. The aetiology of dental caries is presence of plaque and calculus. It is better understood when witnessed rather than when informed. Presence of plaque and calculus is not well untangled to visually impaired people, as it is difficult to explain using disclosing agent. Maintenance of oral hygiene prevents dental caries. However oral hygiene instructions given to normal individuals using visual aids such as tooth brushing models and demonstration of flossing do not well reach visually impaired people.

Blind people fail to recognize dental caries at initial stage such as presence of discolouration or cavity formation. They become aware only after experiencing pain or any discomfort. Negligence in treating caries at initial stage eventually results in loss of teeth. Tooth plays an important role in the oral cavity, loss of which affects the mastication, aesthetic, speech, and even self-confidence. A comparative oral health study between blind and normal school children resulted in 60% prevalence among blind children and only 31.5% in normal school children [5]. In addition, it is common among visually impaired population to prioritise their oral health as less important than the general health and well-being. A Chinese study also found that 92% of visually impaired patients did not have regular access to a dentist, and therefore it was found that 85% of those patients had periodontal pockets, of which 31% percent suffered from deep pockets [6]. Therefore, it is imperative as a public health response to conduct effective oral health promotion programs through adequate oral education and practical demonstration in a way that could help visually impaired individuals to maintain optimum oral hygiene.

Although there are some studies [5] that have explored the oral health of visually impaired population, the information available for visually impaired individuals is still scarce. Therefore, the aim of the present study was to assess the oral health status of institutionalised visually impaired individuals in Chennai city, Tamil Nadu, India.

## 2. Material and Methods

A cross-sectional study was conducted among the visually impaired individuals in Chennai city, Tamil Nadu. According to the 2011 census, Tamil Nadu has 62,538 disabled populations with about 5,583 visually impaired individuals [3]. Chennai city being the capital of Tamil Nadu has 24 shelter homes and school for disabled people, out of which 4 blind schools were selected randomly which were both residential and nonresidential. The list of schools and shelter homes was obtained from blind association of college students and undergraduates, Chennai.

G*∗*Power statistical software was used to estimate the sample size. The sample size required for the study was calculated to be 382, with 80% power and 5% *α* error. The study was conducted among 404 individuals. All blind individuals in the selected institutes between the ages of 15 and 30 were included. Individuals affected with mental retardation, orthopaedic defects, and cerebral palsy and who were physically and mentally handicapped and medically compromised were excluded. The study was presented to the Review Board members and was approved. Following that, the Ethical approval was obtained from the Institutional Ethical committee of Priyadarshini Dental College and Hospital.

The rationale of the study was presented about the purpose of the study to the respective Principals and Head masters of the respected blind institutions and permission was obtained to conduct the study among their students. The students were gathered in a room within the school premises and were individually interviewed about their usual oral hygiene practices. Each student was then clinically examined in the school premises using a halogen light source. The guidelines of the World Health Organization (WHO) were adopted as the diagnostic criteria for dental caries defining a carious tooth as a cavity into the surface of dentine [7]. All the patients' teeth were examined wet, with a ball-ended WHO probe which was employed for examination if necessary. The commonly used indices such as dmft index (for primary dentition) and DMFT index (for permanent dentition) were used and caries prevalence was denoted by the mean number of teeth that were decayed, missing (as a result of decay by extraction) and filled (because of decay) [8].

Following interview and clinical examination, simple oral hygiene education was given highlighting certain practices like mouth rinsing with water after each meal. In addition, brushing technique was also taught by holding the students' hands and demonstrating the correct strokes (Bass method).

All the collected data were analysed using SPSS (Statistical Package for the Social Sciences) version 19 (IBM SPSS Statistics) and the results were obtained.

## 3. Results

**Figure 1:**
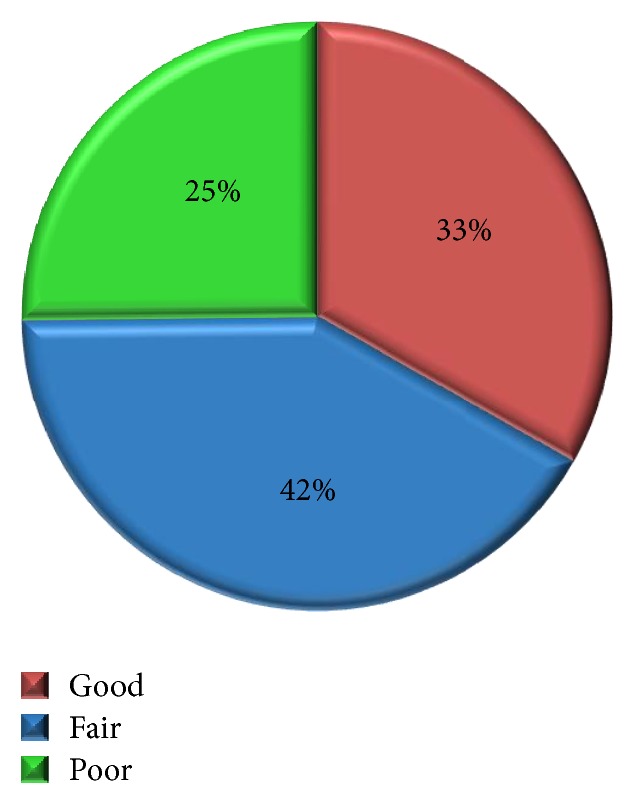
Oral hygiene status of the study subjects.

A total of 404 visually impaired individuals were examined out of which 207 (51.2%) were males and 197 (48.8%) were females. The mean age of the study subjects was 22.63 years.  Figure 1 shows the oral hygiene status of visually impaired individual classified according to the Oral Hygiene Index-Simplified (OHI-S). Whilst 42% of individuals had fair oral hygiene status, 33% had good hygiene followed by 25% having poor oral hygiene

**Figure 2:**
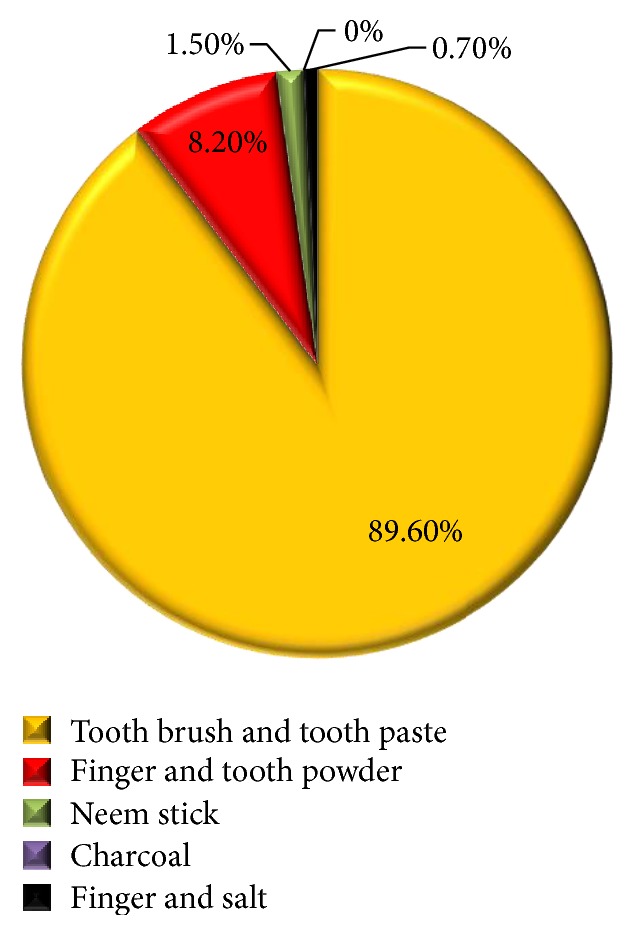
Mode of cleaning the teeth among the study subjects.

The mean number of decayed teeth was 3.1 ± 2.2, mean number of missing teeth was 0.8 ± 1.4, and mean number of filled teeth was 0.5 ± 1.3, in which about 90.3% had decayed teeth, 44% had missing teeth, and only 21% had filled teeth. The mean number of DMFT was 4.5 ± 2.7.  Figure 2 shows the medium used to clean their teeth; the majority used tooth brush and tooth paste; among the individuals examined, 89.6% used manual tooth brush and tooth paste to clean their teeth, whereas 8% used finger and salt as medium for cleaning their teeth.

**Figure 3:**
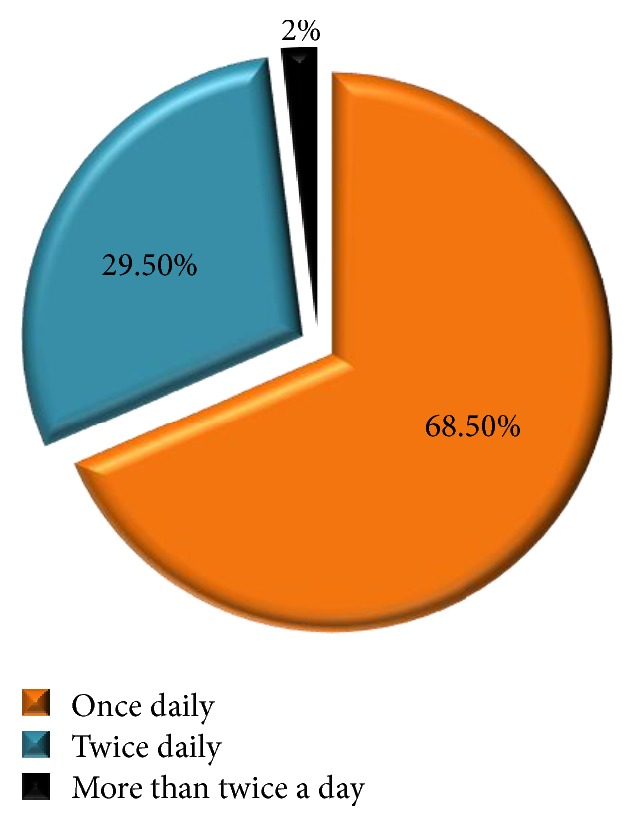
Frequency of brushing among study subjects.

**Figure 4:**
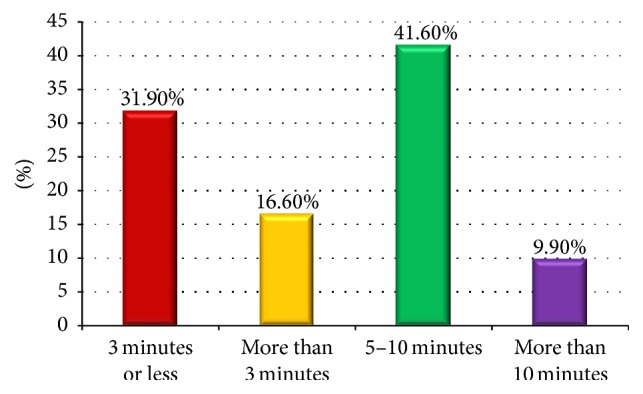
Duration of brushing among study subjects.

**Figure 5:**
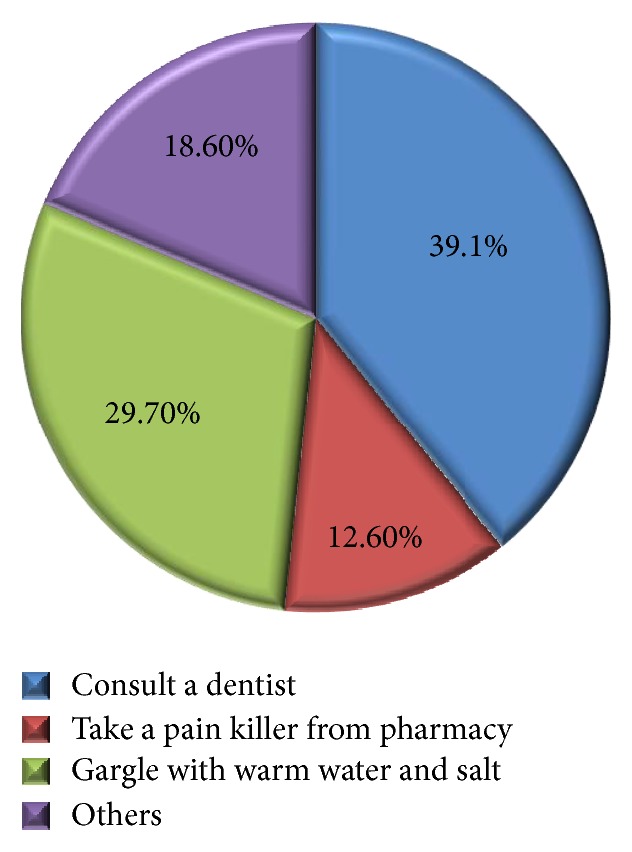
Measures taken during pain by the study subjects.

Figure 3 shows the frequency of brushing their teeth. Whilst a predominant number of sample population (69%) brushed only once daily, only 30% of the students brushed twice daily.  Figure 4 shows the average duration of brushing in the study population. Whilst 43.3% brushed for 5–10 min, 32% brushed for less than 3 minutes per day. The measures taken by the students during dental pain were also clarified during the interview and are shown in Figure 5. It is seen that only 39% of the students consulted a dentist whereas the rest of the population used self-precautionary measures to manage dental pain.

The knowledge, attitude, and awareness among visually impaired individuals about oral healthcare were assessed using the questionnaire which is represented in Table 1. It was observed that a majority of students (70%) performed mouth rinse after meal but only 22% of them used mouth wash. It is also noticed that only half of the sample population (54%) had ever visited the dentist.

## 4. Discussion

The present study evaluated the oral health status, knowledge, and practices of visually impaired students of the four randomly selected blind institutions. Visually impaired people interpret the world around them by echolocation. They tend to acquire knowledge simply by hearing and feeling. A study conducted by Chang and Shih found that students with visual impairments were less knowledgeable about oral care [9]. Contrary to the above statement, the present study shows that the sample population had better knowledge regarding oral health. The young adults in the study were well aware of basic aspects of oral health. This was due to the fact that these students were informed about the importance of oral health by the school teachers through simple oral health education.

In terms of dental visits, previous studies on visually impaired population suggested that a predominant percentage of population had never visited the dentist. For example, the study by Ahmad et al. [10] showed that 92% of the individuals have never visited a dentist whereas another study [11] reported that about 72% had never paid a dental visit. However, the present study shows about 45.3% had never visited a dentist. This can be attributed to the current inclination in the attitude towards dental care. There is a dramatic change in the lifestyle nowadays, which provokes people to be more conscious about their appearance and health.

Improper brushing means and technique are a significant contributor to periodontal problems and other oral diseases. In a study conducted by Singh et al. [12], it is reported that 54% of the visually impaired used tooth brush and paste. In another study by Solanki et al. [4], 74% of the visually impaired used tooth brush and tooth powder, out of which 90.2% of the visually impaired individuals cleaned their teeth once a day, whilst only 0.9% cleaned their teeth twice. Both the above studies stated that blind individuals had improper brushing means, which highlight the lack of knowledge about effective use of tooth brushes with dentifrices. The present study shows a positive result of 86.9% of students using tooth brush and tooth paste to clean their teeth and only 8.2% using tooth powder with fingers. In addition, a majority of the participants (68.6%) brushed at least once a day and 29.5% brushed twice. This clearly states that there is more room for promoting awareness for not only the effective use of tooth brush and tooth paste, but also the proper techniques of tooth brushing.

The present study reported that 49% had experienced tooth sensitivity and 48% had oral malodour. Similar findings have been reported by another study [13] where 58.3% had experienced tooth sensitivity and 30.8% oral malodour. Blind individuals have a sharp sensation, which helps them in identifying certain conditions such as sensitivity and malodour, the presence of which indicates periodontal problem. Additionally, the use of mouthwashes was observed to be very low among the participants. Similar findings have been observed across other studies regarding the use of supplementary oral hygiene materials such as mouthwashes and dental floss [13, 14]. The use of floss has been neglected as it is difficult to demonstrate the technique to visually impaired individuals. The limited usage of mouthwashes may be because of lack of knowledge or due to the probability of misinterpreting with any other toxic solution.

Oral hygiene of an individual has an essential part in the oral health status. The study shows that 70% of the people in present study rinsed their mouth after each meal, which contributes to the basic method of clearing food debris from the mouth. This finding is consistent with a study conducted by Prashanth et al. which reported that the oral hygiene was good for 91.76%, fair for 5.88%, and poor for 2.35% for blind individuals [15]. The present study shows that the hygiene status was fair for most being 42% and 33% having good hygiene followed by 25% having poor oral hygiene. When comparing all the above studies it is shown that hygiene status varies from person to person as in normal individuals. Proper technique to maintain oral hygiene has not been effectively communicated to handicapped people as in normal individuals. Presences of plaque and calculus may not be well disclosed among the visually impaired using disclosing agents; thus the necessity remains untold.

The rate of dental caries has been reported to be higher among the disabled population in comparison to normal population for all age groups. The present study reported a high prevalence of caries 90%, of which the largest component of DMFT was the decayed teeth index which was estimated to be 3.14 on average and the filled index had the least mean score of 0.54. The findings of high caries rate and DMFT scores are comparable to other studies [12, 16]. When compared to the above studies dental caries increase with age. With increase in caries prevalence and severity with increasing age, this finding was attributed to the irreversibility and accumulative nature of the disease with age [17]. Various reasons can be put forward to decipher intensity of dental caries, such as biochemical differences in salivary buffering to differences in living environment, dietary and habits, different proportions of salivary components, and possible differences in chemical composition of the saliva. Basic preventive measures such as topical fluoride application, pit, and fissure sealant are not initiated in these students. In addition, parents of blind individuals fail to recognize the importance of early dental care.

The highlights of the study include a healthy amount of sample population with focus on the transitional age groups (teenage to young adult age groups) who were examined to gain insights about prevalence of caries unlike other studies which focus only on children. Presence of a relatively high caries rate when compared with other studies leaves an alarming state for treatment need. The limitation in the study was that the other sociodemographic and sociocultural predictors for oral hygiene were not accounted. The use of DMFT index among adults could be misleading as adults may have lost teeth for reasons other than dental caries. In addition, questions regarding dietary practices among the sample population were not included in the study, which could have added more value in regard to their oral hygiene status. Future scope includes incorporation of custom designed health promotion methods such as braille and/or music-based oral hygiene programs which would encourage active participation and helps promote better oral healthcare towards visually impaired people.

## 5. Conclusion

As per the present study, whilst it is evident that visually impaired individuals have fair oral hygiene status, they lack knowledge about proper brushing techniques which was one of the reasons for high rate of dental caries. In order to amend their condition, suitable preventive measure such as comprehensive dental care are an important need. Whilst oral health awareness is provided through media, it is imperative that these students be taught practically through biannual oral health promotion camps and sequential screening assessments. Early identification of caries and proper guideline for maintenances of oral hygiene should be well informed to parents, guardians, and school teachers. Primary health centres are to be developed for the welfare of disabled people in order to promote their oral and general health.

## Figures and Tables

**Table 1:** Knowledge, attitude, and awareness of oral health among sample population.

S. number	Questions	Yes *N* (%)	No *N* (%)
(1)	Do you rinse your mouth with water after each meal?	283 (70)	121 (30)
(2)	Do you use mouth wash?	88 (21.8)	316 (78.2)
(3)	Have you felt any sensitivity in your teeth before, while having cold or hot drink?	198 (48)	206 (52)
(4)	Have you experienced any bad smell while talking?	194 (48.1)	210 (51.9)
(5)	Have you ever visited a dentist before?	221 (54.7)	198 (45.3)
